# Mr. Wadley, on Venæsection

**Published:** 1805-10-01

**Authors:** Thomas Weston Wadley

**Affiliations:** Wotton Basset


### *To the Editors of the Medical and Physical Journal.*

GENTLEMEN,

A Country Practitioner has seldom much leisure to notice on paper the theories or remarks of others; some strong expressions of the Physician to the Finsbury Dispensary, in your last Number, have however induced me to offer a few remarks on them.

With the greatest deference to the judgment of Dr. Reid, I must take the liberty of differing from him in opinion on the subject of venæsection, not when he says that it is very rarely advisable in "this enervated age," but in the *degree* of *fatality* occasioned by an improvident use of the lancet. I am by no means an advocate for frequent blood-letting, nor have I a wish to *carp* at an unguarded expression of any of your correspondents, knowing how much I myself stand in need of their candour; but when it is positively affirmed by a physician to a public institution, that "if the employment of the lancet were abolished altogether, it would, perhaps, save annually a greater number of lives than in any year the sword has ever destroyed;" junior practitioners might be lost between the rules of authors and the *ipse dixit* of the Reporter. This is not all, your widely extended and valuable Journal is read by numbers not of our profession, and what must *they* think of medical practice after reading the above quotation?

Superstition

Superstition, or at least timidity, is a frequent attendant on the bed of disease, and it is not impossible or improbable but that many a valuable life might be lost from non-compliance with the wishes of the medical attendant who advises venæsection; his patient might have seen what comes from so high an authority, and knowing that the *sword* has (unhappily) of late years sent *millions* of our fellow creatures prematurely "to that bourne from whence no traveller returns," he might recollect Dr. Reid's comparison, and actually fall a victim to his fate, for want of that remedy he feared would only hasten it. If Dr. Reid, when he insinuates so much mischief has been done by the lancet, alluded to the operations of those *appendages* to the profession who are to be found in every country village, under the denomination of barbers, bone-setters, and farriers, he is in some degree right, no surgeon in much practice but must have had the mortification to see frequent instances of the abuse of the lancet by these people, and to see every means fail that art could devise, to restore the accustomed tone to a debilitated system; but in regularly educated men of some experience, surely the fatality caused by the lancet in their hands is not quite so extensive; indeed, I believe that practitioners are so well aware of the Doctor's remark, "that inflammatory complaints are seldom to be met with," (at least in this country) that very few indeed use the lancet once in one hundred cases of fever.

I have read your Journal, Gentlemen, from the commencement of its publication, and have as constantly seen with peculiar pleasure the remarks of the Reporter of the Cases at the Finsbury Dispensary, and still hope to see from time to time the productions of his elegant pen; and as he was pleased to qualify an expression in a former number, wherein it was said, "unless the constitution has been exhausted either by time or intemperance, no one *ought* to die of fever," so possibly he will, on reflection, think the writer of this article not actuated by any other motive than the regard he has for the welfare of the community and the honor of the medical profession, and will be induced to think somewhat differently of the extent of the injury inflicted by venæsection.

I am, &c.

THOMAS WESTON WADLEY.

*Wotton Basset, Aug.* 10, 1805.

